# Supplementary material for: Can spatial filtering separate voluntary and involuntary components in children with dyskinetic cerebral palsy?
Source: PLoS One. 2021 Apr 14;16(4):e0250001. doi: 10.1371/journal.pone.0250001 (PMC8046213; doi:10.1371/journal.pone.0250001)
Supplement: Attachment — Submitted filename: Response to Reviewers v2.docx [file pone.0250001.s003.docx]

**We are very grateful to the anonymous Reviewers and the Editor for the many productive comments and suggestions. In the revised manuscript, we believe we have addressed all their concerns.**

**Response to the Reviewers**

Reviewer #1: This is a highly relevant, well conducted and articulated research.

**We are grateful to the Reviewer for the valuable comments and suggestions. We believe that, by addressing the Reviewer’s concerns, the revised discussion has significantly improved.**

There is concern regarding the multi-phase nature of this device control study, that extends the application of results to reveal movement mechanisms underlying dyskinetic CP, leading to over-interpretation of results. This is a complex study in a very small sample and these limitations need to be recognized. There are many potentially confounding variables at each phase of the study i.e., other explanations, that would change implications for the movement mechanisms underlying dyskinetic CP. The implications for device myo-control are appropriately discussed.

To determine if identifiable dyskinetic movement patterns exist and imposed on/within voluntary movements in dyskinetic CP, movement profiles and EMG patterns can be and are analyzed in detail. These results have direct implications on movement mechanism underlying dyskinetic CP and should be discussed.

Further phases of the study i.e., elimination of muscles/ EMG components and use of the device myo-control, introduce too many confounds to have meaningful implications for movement mechanism. These results do have direct implications for device control strategy and myo-control performance. I recommend eliminating the sentence in the Abstract and on p.27 regarding, ‘This work provides insight in where the noise lies…’ and contain comments on movement mechanism to results of the first phase of the study.

**We rephrased the discussion in several parts to address the Reviewer’s concerns:**

**“*Our findings further confirm the results reported in previous studies suggesting that dystonia in dyskinetic CP is characterized by an impaired ability to properly suppress variable and task uncorrelated muscle activity [12,44]. Both primary and secondary dystonia have been associated with injury or dysfunction in the basal ganglia. It has been suggested that abnormalities arising from basal ganglia malfunction in dystonia may be related to the inability to remove unwanted components of movement [45,46]. Therefore, motor impairments in dyskinetic CP could represent the superimposition of unwanted muscle activations on the desired movement pattern, rather than additional or differing structures of motor modules during the motor execution [42, 47]. Moreover, it has been suggested that hyperkinetic and unwanted muscle activations in upper extremities in children with dyskinetic CP are characterized by increased signal-dependent noise of the motor system [13, 14,15, 24]. This means that children dyskinetic CP in our study would have also required significantly slower movements to contain the increased motor variability and resulting in poorer controllability of myocontrol [41].*”**

**“ … *A study done on adults showed different adaptation rates in a myocontrol task depending on the span of the synergies [38]. For children with dyskinetic CP, projecting to a synergy space different from their own may filter noise from some dimensions not necessary for control while allowing participants to learn to control a lower dimensional space. Future work would be needed to determine whether the exploitation of learning environment by removing or manipulating specific synergies components or muscles could promote functional synergy recruitment and synergy reorganization resulting with a better myocontrol performance.*”**

***“ … Our study has some limitations that will need to be addressed in future research. A larger cohort would be needed to derive final conclusions on the effects of filtering to extract the relevant components to improve myocontrol interfaces in children with dyskinetic CP. Subject 2 was characterized by mild symptoms of dystonia (i.e. low rating of BAD scale), and performed the myocontrol task with the highest success rate compared to the other subjects. This suggests that the exacerbation of motor impairments increases the extent to which the unwanted components muscle activations interfere with the voluntary components during myoelectrical activity. Therefore, it would be worthwhile to further investigate how severity of motor impairments in dyskinetic CP effects the filtering of controllable components on EMG control performance.*”**

***“… We have learned that selecting the most controllable muscles or synergies as quantified by Fitts’ Law does not improve myocontrol performance for children with dyskinetic CP, and can make performance even worse in some subjects (e.g. higher success rate for Subject 1 with the All Muscle condition). This appears to result from the spread of controllability and unwanted muscle activations over all available dimensions of control. As a result, the involuntary movement components are not confined to a low-dimensional subspace, and they cannot be removed by subspace filtering …*”**

Reviewer #2: The work reports clearly describes an interesting study. However, some clarifications and further details are needed.

Experimental protocol

• Were the 2 force-control blocks carried out before each of the 3 conditions?

**Yes, they were. We added the information in the text:**

**“*On each day, participants initially performed two blocks of trials in force-control.*”**

• It is not clear the goal of the first force-control block. Is to estimate subject-specific MVF? And then to compute MT? Therefore, wasn’t MT computed during the 240 trials for each condition?

**The goal of the first force-control block was to estimate the subject-specific MVF. The MT during the first force-control block was not used in the performance analysis.**

**The request to perform “as quickly as possible” was to compel the participants with a high rate of force development to assess the MVF.**

• Was the second force-control block used to compute H and ef (from equation 2)? It might be explicitly linked in the text

**Yes, it was. We specified in the “EMG-to-force mapping” paragraph:**

**“*The EMG-to-force matrix (H) was estimated using multiple linear regressions of each applied force component with EMG signals recorded during the second force-control block.*”**

• MT was set at 7.5 seconds as maximum, how was this value defined?

**The limit at 7.5 seconds to reach at the target was defined based on pilot data testing and allowed to provide enough time to complete the task by considering the possibility that involuntary sustained or intermittent muscle contractions due to dystonia could occur during the performance.**

**If an unsuccessful trial occurred, the participant immediately repeated the trial. Only successful trials were considered in the analysis**

**Moreover, it is worthwhile to note that the average MT for all subjects ranged from 2.08 to 3.55 seconds.**

**We better specified in the text:**

**“*Participants initially performed two blocks of trials in force-control. In the first force-control block, the mean maximum voluntary force (MVF) along eight directions (separated by 45°) (Fig 1) was estimated as the mean of the maximum force magnitude recorded across 16 successful trials (two for each direction) in which participants were instructed to generate maximum force in each direction.*”**

**“*For unsuccessful trials, MT was set at 7.5 seconds, which was the maximum trial length. If an unsuccessful trial occurred, the participant immediately repeated the trial. The limit set at 7.5 seconds was defined based on pilot data testing and allowed to provide enough time to complete the trial by considering the possibility that involuntary sustained or intermittent muscle contractions due to dystonia could occur during the performance.*”**

• “widths 4.31, 7.24, and 12.18 % MVF, resulting in IDs equal to 1.3, 2.05, and 2.8 bits respectively..” Shouldn’t ID and W be inversely “proportional”?

**We apologize for the typos; we inadvertently inverted the order of the IDs.**

**We corrected the text:**

**“*Targets corresponded to a distance of 15 % MVF from the origin with widths 4.31, 7.24, and 12.18 % MVF, resulting in IDs equal to 2.8, 2.05 and 1.3 bits respectively, and were presented in random order.*”**

• Why is there a different range between force-control block and “Select Muscle” and “Select Synergy” condition blocks? 0-50% MVC and 0-25% MVC

**It is worth noting that 0-50% MVF and 0-25% MVF refer to the range of cursor position from the midpoint to the edge of the monitor screen.**

**In the second force-control block we chose the 0-50% MVF range screen in order to display targets until 30% MVF, since for this condition a larger range of muscle activations was needed for mapping computation.**

**In the “Select Muscle” and “Select Synergy” conditions we selected a range 0-25% MVF since the largest muscle activation was 12.18% of the MVF.**

***“In the second force-control block, participants performed 24 trials to targets positioned at force magnitudes corresponding to 10, 20, and 30 % MVF (random order within cycles of eight directions) with a target width (diameter) corresponding to 5 % MVF, presented in random order. The cursor position was linearly proportional to the EMG such that 0% MVF was the midpoint of the screen and 50% MVF was the edge of the screen.”***

**“*For the*** ***Select Muscle and Select Synergy conditions …	Targets corresponded to a distance of 15 % MVF from the origin with widths 4.31, 7.24, and 12.18 % MVF …	For Select Muscle and Select Synergy conditions the cursor position was linearly proportional to the EMG such that 0% MVF was the midpoint of the screen and 25% MVF was the edge of the screen*”**

• Why was the movement onset in some cases considered as 5% of peak velocity and in other cases the time when the target appears?

**We are grateful for pointing this out, we are aware that in the “EMG-to-force mapping” section these some improper definition of the timing of the dynamic phase. MT was calculated as the time from when the cursor velocity was greater than 5% peak velocity during the trial to the time once they successfully stabilized in the target for 0.2 seconds.**

**We better explained in text:**

**“*Movement time (MT) was calculated as the time from when the cursor velocity was greater than 5% peak velocity during the trial (t0) to the time once they successfully stabilized in the target for 0.2 seconds*”**

**“*The EMG-to-force matrix (H) was estimated using multiple linear regressions of each applied force component with EMG signals recorded during the second force-control block. Only data from the dynamic phase of trials were used for regression, from t_0_ until the cursor was stabilized in the target for 0.2 seconds*.”**

**“*Muscle synergies were identified by Non-negative Matrix Factorization (NMF) from EMG patterns during the force control block using data from t_0_ until the participant stabilized the cursor within the target for 0.2 seconds (dynamic phase).*”**

Data analysis

Not very clear the paragraph about the success rate. Later in results, in Table 2, the values are from 0 to 1, as proportion of successful trials. How are they linked?

**The table 2 shows the success rate. We ran the statistical analysis on odd ration instead where the outcome is either 1 or 0 (they hit the target or didn’t), rather than relying on the aggregate results (e.g. success rate). We clarified the text in the “Data Analysis” section as it follows:**

**“*In order to compare success rate, we computed the odds ratio for the statistical analysis rather than comparing an average success over N trials. This resulted with values 0 or 1 depending on whether participants missed or hit the target. With odds ratio higher than 1 success is more likely than failure; less than 1 failure is more likely than success; and equal to 1 success and failure are equally likely.*”**

Results

From Fig 2

• Isn’t there any repeatability of planar force amplitude neither of pull direction generated by each muscle (row A), across the 5 subjects?

**We agree with the Reviewer, and the concern was addressed in the discussion:**

**“*The H matrices and force pulling directions of muscles were inconsistent among the participants. This could be caused by involuntary sustained muscle contractions and co-activations between agonists and antagonists, which may result in in different pulling force direction respect to the expected ones. However, even if the pulling directions were not estimated very accurately, they were used for both muscle and synergy control, and therefore marginally affect the comparison between control conditions.*”**

• To clarify the muscle selection: “the 4 muscles with highest TP that also spanned the 2-D space”. They are not the most involved in row A (e.g. m 2 of subj. 5 in row A bigger than muscle 7, but muscle 7 is selected in row B)

**The selection of the muscles and synergies was based on the TP assessment, which allowed to select the less controllable ones.**

**Please, refer to the text in the methods:**

**“*The dimensionality of the muscle and synergy mappings was then reduced by eliminating less controllable muscles or synergies. The 4 muscles and synergies with the highest TP were selected for the 2-D task. The criterion for selecting the synergies was based on a previous study showing that in healthy subjects the trajectories reconstructed using 4 synergies were as accurate as the trajectories estimated from EMGs recorded from the entire set of muscles [27].*”**

• Row B: Subj 3 changed colour (e.g. muscle 4 from green in row A to light blue in row B), what does it mean?

**An incorrect color for muscle 4 on row B, Subj 3 was used. We corrected the Figure by changing light blue with green color.**

• The number of synergies is free? 5 6 or 7 synergies for the different subjects

**As described in in the text, “*For each participant the number of synergies adequately capturing the EMG data (N) was selected according to the fraction of data variation explained, defined as***

$$\boldsymbol{R}_{\boldsymbol{EMG}}^{\boldsymbol{2}}\boldsymbol{=1-}\frac{\boldsymbol{SSE}_{\boldsymbol{EMG}}}{\boldsymbol{SST}_{\boldsymbol{EMG}}}$$

***where SSE_EMG_ is the sum of the squared muscle activation residuals and SS_TEMG_ is the sum of the squared residuals of the muscle activation from its mean vector. We extracted a minimum of five synergies and additionally considered two criteria. The first criterion was a threshold of 0.9 on R^2^EMG. The second EMG criterion was based on the detection of a “knee” in the slope in the curve of the R^2^ value as a function of N. A series of linear regressions was performed on the portions of the curve included between N and its last point (M). N was then selected as the minimum value for which the mean squared error of the linear regression was less than 10^−4^ [27,38].”***

**Based on these 2 computational criteria subjects could theoretically result with varying number of synergies (as it resulted in our studies indeed), then the less controllable synergies were removed for synergy-control condition (see response to the comment below)**

• How the 4 synergies are selected? always 4? Aren’t they the ones producing higher force (e.g. subj 3, synergy 7 (mainly BIC) is bigger than synergy 4 (mainly PD), but synergy 4 is selected)

**Synergies were ranked based on average information throughput (TP) computed through the 1-D isometric speed-accuracy task. The dimensionality of the synergy mappings was then reduced by eliminating less controllable synergies. The four muscle synergies with the highest throughput were selected for the 2-D task.**

**The rationale for choosing 4 synergies for the synergy-control condition derives from a previous study (Berger & d’Avella, 2014) showing that trajectories reconstructed using 4 muscle synergies were as accurate as the trajectories obtained from the estimation of considering all the set of muscles in healthy subjects.**

**We better explained in the text:**

**“*The dimensionality of the muscle and synergy mappings was then reduced by eliminating less controllable muscles or synergies. The 4 muscles and synergies with the highest TP were selected for the 2-D task. The criterion for selecting the synergies was based on a previous study showing that in healthy subjects the trajectories reconstructed using 4 synergies were as accurate as the trajectories estimated from EMGs recorded from the entire set of muscles [27].*”**

• The two synergies cited above are even quite parallel; do BIC and PD produce similar planar forces?

**As stated above the synergies extracted for each subjects were ranked based on the average information throughput (TP) computed by means of the 1-D isometric speed-accuracy task. Note that the movement was constrained to one axis in the pulling direction synergy. The synergies with the highest 4 TP were than selected for the synergy control condition. Moroever, If the pulling direction of a muscle or synergy made an angle of 60° or less with that of a muscle or synergy with higher throughput, then we did not select it in order to ensure that the selected muscles and synergies spanned the 2-D space.**

**Please refer to the “Muscle and synergy selection” paragraph, here are reported the relevant details in the text:**

**“*To select the muscles and synergies to be used in the 2-D myocontrol task, participants performed a 1-D isometric speed-accuracy task….***

***… The movement was constrained to one axis in the pulling direction for either each muscle and synergy.***

***… For each synergy or muscle, participants completed 40 trials, reaching to four targets of different widths (3.0, 4.3, 6.1, 8.6 % MVF) and distances (7.5 and 15 % MVF) in each component’s natural pulling direction calculated from the EMG-to-Force mapping (Fig 2, Row A and Row C). IDs for the 1-D task ranged from 0.80 to 3.32 bits.***

***… The dimensionality of the muscle and synergy mappings was then reduced by eliminating less controllable muscles or synergies. The four muscle synergies with the highest throughput were selected for the 2-D task***

***… The dimensionality of the muscle and synergy mappings was then reduced by eliminating less controllable muscles or synergies. The four muscle synergies with the highest throughput were selected for the 2-D task.***

***… If the pulling direction of a muscle or synergy made an angle of 60° or less with that of a muscle or synergy with higher throughput, then we did not select it in order to ensure that the selected muscles and synergies spanned the 2-D space (Fig 2, Row B and Row D). The angular difference between two muscles or synergies was calculated as the inverse cosine of their pulling directions.*”**

From Fig 3

• Is there any order/hierarchy of synergies based on explained variance?

**It is worthwhile noting that for NMF there is not a given fraction of variance associated to each synergy as in PCA. However, it is possible to assess the contribution of each synergy by comparing the R^2^ achieved with the full set with the R^2^ with the set without the synergy being assessed.**

• Which is the 1-D task used to select the synergies (in black)? Why not using the 2-D task itself?

**The synergies indicated in black colors were the 4 synergies selected from the 1-D isometric speed-accuracy task. This allowed to constrain the movement in only one direction and to assess each muscle and synergy individually.**

**We edited the text to better clarify:**

**“*The movement was constrained to one axis in the pulling direction for either each muscle and synergy. This allowed to constrain the movement in only one direction and to assess each muscle and synergy individually.*”**

About fig 4:

• It seems that participant 2 shows MT for Select Muscle also lower than MT in All EMG, isn’t it?

**We are very grateful to the Reviewer to pointing this out. We erroneously reported the same MT of All Muscle condition. We corrected Figure 4**

• Subjects 1 and 4 seem to have “significant” higher MT in All EMG, isn’t it?

**The statistical analysis did not find significant differences between conditions in subjects 1 and 4, which as mainly due to a large data distribution within the subjects. It is worthwhile noting that the fact that two SEM error bars do not overlap does not let make any conclusion about statistical significance.**

About odds ratio.

• When reporting 0.44, or 0.30 in the text, are they averaged across subjects?

**Yes, they are. We specified in text:**

**“*The odds ratio associated with an increase in ID was on average 0.44 across all subjects,***

***… with Select Muscle having an average odds ratio of 0.44 compared to All EMG …***

***… with Select Synergy having an average odds ratio of 0.30 compared to All EMG (p < 0.0001).*”**

• Table 2. Subject 1 in “All EMG” has a very higher success rate than in the other 2 conditions. But this could be due to a slower movement in All EMG. It might be discussed.

**Thank you for bringing it up. There was no statistical difference on MT among conditions in subject 1. However, it might be possible that selecting the most controllable muscles or synergies led to worsen myocontrol performance, which could result from the spread of controllability and unwanted muscle activations over all available dimensions of control.**

**We discussed in the manuscript as it follows:**

**“*This study sought to determine if spatially filtering EMG from children with dyskinetic CP would improve myocontrol performance. We have learned that selecting the most controllable muscles or synergies as quantified by Fitts’ Law does not improve myocontrol performance for children with dyskinetic CP, and can make performance even worse in some subjects (e.g. higher success rate for Subject 1 with the All Muscle condition). This appears to result from the spread of controllability and unwanted muscle activations over all available dimensions of control. As a result, the involuntary movement components are not confined to a low-dimensional subspace, and they cannot be removed by subspace filtering*.”**

• Is there any correlation between severity and outcome measure? E.g. the less severe subject (subj 2 with BAD 1 on tested arm) seems to have highest success rate

**We did not find any correlation between the BAD scale and the performances (as it can be seen by comparing Table 2 with BAD scale of all subjects). However, it is quite possible that the high rate of success in subject 2 is due to mild symptom of dystonia. We reasoned this result in the discussion and the study limitation section:**

**“*Our study has some limitations that will need to be addressed in future research. A larger cohort would be needed to derive final conclusions on the effects of filtering to extract the relevant components to improve myocontrol interfaces in children with dyskinetic CP. Subject 2 was characterized by mild symptoms of dystonia (i.e. low rating of BAD scale), and performed the myocontrol task with the highest success rate compared to the other subjects. This suggests that the exacerbation of motor impairments increases the extent to which the unwanted components muscle activations interfere with the voluntary components during myoelectrical activity. Therefore, it would be worthwhile to further investigate how severity of motor impairments in dyskinetic CP effects the filtering of controllable components on EMG control performance.*”**

About TP.

• SEM: how is it defined? (in Fig 5 Standard Error (SE) is reported and cited in caption)

**We corrected the standard error of the mean (SEM) in the figure captions.**

• Subjects 1 and 4 seem to have “significant” higher TP in Select Muscle than in All EMG, isn’t it?

**As stated above, please note that if two SEM error bars do not overlap does not let make any conclusion about statistical significance.**

Discussion

• The first claim “We found that Select Synergy and Select Muscle myocontrol had higher movement time and lower success than All EMG myocontrol” doesn’t seem to be strongly supported by data.

**We apologized for reporting improper interpretation of the results. We better clarified as it follows:**

**“*The experimental task was designed using the Fitts’ Law paradigm to test the controllability of components identified by synergy decomposition and selected muscles. A linear relationship was found between the MT to achieve the target and the ID, as expressed by the Fitts’ model, for all the experimental conditions (i.e. All Muscle, Select Muscle and Select Synergy). The findings are in accordance with previous studies reporting that myocontrol performances followed the Fitts’ Law in healthy control subjects [33,34,37] and patients with dyskinetic CP and primary dystonia [41]. However, the overall results did not show significant changes in performance for Select Muscle and Select Synergy compared to All Muscle condition either for MT, TP and success rate.*”**

• It may be better explained how a different synergy space could improve control through a more effective learning

**We added further explained in the context of learning:**

**“*For children with dyskinetic CP, projecting to a synergy space different from their own may filter noise from some dimensions not necessary for control while allowing participants to learn to control a lower dimensional space. Future work would be needed to determine whether the exploitation of learning environment by removing or manipulating specific synergies components or muscles could promote functional synergy recruitment and synergy reorganization resulting with a better myocontrol performance.*”**

• The main conclusion is that the EMGs of CP children are overall corrupted by noise. It should be better discussed if it was already a proposed theory in previous works.

**The topics concerning the noise and involuntary muscle components were further discussed:**

**“*Our findings further confirm the results reported in previous studies suggesting that dystonia in dyskinetic CP is characterized by an impaired ability to properly suppress variable and task uncorrelated muscle activity [12,44]. Both primary and secondary dystonia have been associated with injury or dysfunction in the basal ganglia. It has been suggested that abnormalities arising from basal ganglia malfunction in dystonia may be related to the inability to remove unwanted components of movement [45,46]. Therefore, motor impairments in dyskinetic CP could represent the superimposition of unwanted muscle activations on the desired movement pattern, rather than additional or differing structures of motor modules during the motor execution [42, 47]. Moreover, it has been suggested that hyperkinetic and unwanted muscle activations in upper extremities in children with dyskinetic CP are characterized by increased signal-dependent noise of the motor system [13, 14,15, 24]. This means that children dyskinetic CP in our study would have also required significantly slower movements to contain the increased motor variability and resulting in poorer controllability of myocontrol [41].*”**

Few typos, e.g.

o “postion”,

**Corrected**

o TP acronym used before definition

**We corrected as it follows:**

**“*Muscles and synergies were then ranked based on average information throughput (TP), which is defined as the quantitative measure of controllability in terms of bit rate [32]*:”**

o Fig 2 caption: reference to Fig2 itself, I think it should be Fig3

**Corrected**

**Response to the Editor**

https://doi.org/10.1016/j.ijhcs.2020.102432. Specifically the paragraph in the introduction of your submission which begins: 'Fitts’ Law is the well-known mathematical formulation....'

In your revision please rephrase any duplicated text - further consideration is dependent on these concerns being addressed.

**We rephrased as requested:**

**“*Fitts’ Law is a well-established model for the trade-off between speed and accuracy [29], who showed that the movement time (MT) to achieve a target in proportional with increased movement distance (D) and decreased target size (W). The Fitts’ Law was derived from the information theory by Shannon [30] and adapted to the human motor system. Given its direct linkage with information theory and the channel capacity theorem, Fitts's Law has effectively applied in the field of human-computer interfaces (HCI) as a tool to maximize the information rate for a given device, such as mice and touchscreens [31, 32]. Therefore, Fitts's law proves to be also a valuable tool to assess controllability of myocontrol interfaces [33-37].*”**

Please upload a copy of Figure 8, to which you refer in your text. If the figure is no longer to be included as part of the submission please remove all reference to it within the text.

**We are sorry for the misunderstanding in discussion. In the phrase “In a study comparing reaching and figure 8 drawing movements, children with dystonia and typically developing children shared many similar synergies, however time activations differed [41]”, we refer to a previous study (Lunardini et al., 2017) in which subjects were asked to perform a “Figure 8” drawing task. We edited the text to avoid any confusion.**

We note that you have indicated that data from this study are available upon request. PLOS only allows data to be available upon request if there are legal or ethical restrictions on sharing data publicly. For more information on unacceptable data access restrictions, please see http://journals.plos.org/plosone/s/data-availability#loc-unacceptable-data-access-restrictions.

**We uploaded the data on the Supporting Information files section.**
